# A Comparative Study of Flash Nanoprecipitation and Sequential Nanoprecipitation: Impact of Formulation Parameters on Drug-Loaded Nanoparticle Formation

**DOI:** 10.1021/acs.molpharmaceut.5c00835

**Published:** 2025-09-17

**Authors:** Nouha El Amri, Amy McKinstry, Rachel E. Pollard, Parker K. Lewis, Nathalie M. Pinkerton

**Affiliations:** † Department of Chemical and Biomolecular Engineering, Tandon School of Engineering, 34242 New York University, Brooklyn, New York 11201, United States; ‡ Department of Biomedical Engineering, Tandon School of Engineering, New York University, Brooklyn, New York 11201, United States

**Keywords:** drug delivery, nanoparticles, flash nanoprecipitation, sequential nanoprecipitation, continuous flow manufacturing

## Abstract

Flash NanoPrecipitation (FNP) is a well-established method for forming core–shell drug-loaded nanoparticles that has demonstrated effectiveness for encapsulating highly hydrophobic compounds. However, certain formulation challenges persist, including size limitations and reduced encapsulation efficiency for moderately hydrophobic drugs. Sequential NanoPrecipitation (SNaP) is a novel nanoparticle synthesis process that relies on the same assembly principle as FNP, while decoupling the core formation and stabilization. Through a systematic comparison using drugs with varying hydrophobicity, we demonstrate complementary capabilities between these techniques. We showed that for the high hydrophobicity drugs such as β-carotene, both SNaP and FNP performed effectively. In the case of cinnarizine, a moderately hydrophobic drug that requires the use of hydrophobic ion pairing, good encapsulation efficiency was observed for the case of SNaP, while no encapsulation was observed with FNP. For ibuprofen, a water-soluble analgesic, low encapsulation efficiency was observed with SNaP, and no loading was observed with FNP. Release studies with itraconazole nanoparticles revealed that SNaP-produced nanoparticles exhibited approximately 50% slower release rates compared to FNP over 48 h. Additionally, SNaP enabled access to a broader size range and successful nanoparticle formation at lower solid concentrations. These findings establish SNaP as a valuable complement to FNP, particularly for applications requiring larger nanoparticles, encapsulation of moderately hydrophobic compounds, or modified release kinetics. Together, these nanoprecipitation approaches provide formulators with expanded capabilities for developing polymeric nanoparticle drug delivery systems.

## Graphical abstract

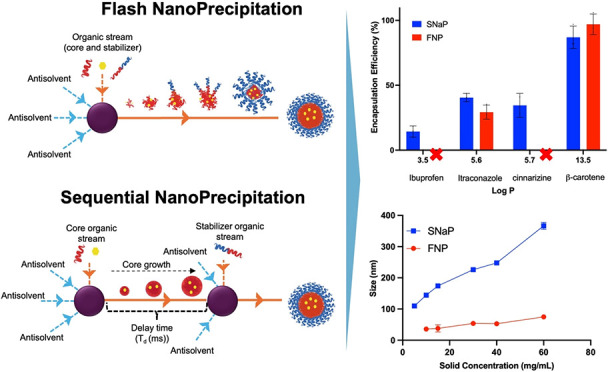

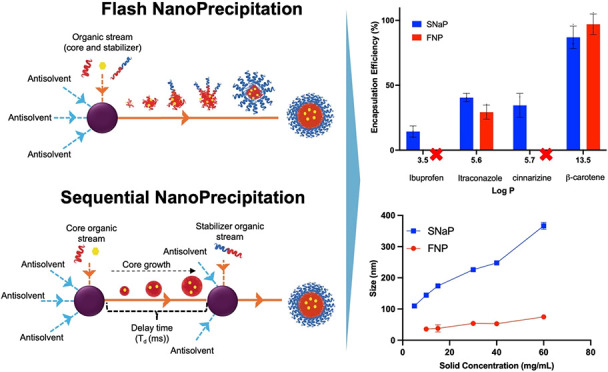

## 1 Introduction

Polymeric nanoparticles have emerged as powerful vehicles for drug delivery, offering enhanced bioavailability, improved stability, and targeted delivery capabilities.^1,2^ Despite their promise, clinical translation remains limited due in part to challenges in scaling synthesis processes while maintaining control over particle characteristics.^3^

The Flash NanoPrecipitation (FNP) process is a scalable, one-step process for forming core–shell polymeric nanoparticles by inducing controlled precipitation through turbulent mixing.^4,5^ Nanoparticle formation is achieved via diffusion-limited self-assembly driven by hydrophobic interactions.^6,7^ In FNP, a water-miscible organic stream containing hydrophobic bioactive molecules (e.g., therapeutics and/or fluorophores) and amphiphilic block copolymers (BCP) is rapidly mixed against antisolvent aqueous streams (Figure 1A).^8,9^ This induces homogeneous supersaturation of components, leading to the rapid nucleation and growth of the hydrophobic molecules to form the nanoparticle core.^8,10^ The particle growth is arrested when the hydrophobic block of the BCP adsorbs onto the core and the hydrophilic block sterically stabilizes the nanoparticle, forming a shell.^11,12^ The self-assembly process takes 15–50 ms.^11^ FNP typically employs confined impinging jet mixers (CIJM) or multi-inlet vortex mixers (MIVM) operating at high flow rates (*Re* > 1600) to generate a micromixing environment with mixing times below 5 ms.^13–16^ The rapid micromixing is required to ensure homogeneous supersaturation of the components prior to assembly. The FNP process enables control over particle size and composition and can achieve high drug loadings and encapsulation efficiencies, making it highly suitable for applications in drug delivery.^4,17^ However, three major limitations remain: the particle size is generally restricted to below 300 nm,^18^ encapsulation of inorganic components (e.g., quantum dots, iron oxide nanocrystals) is not uniform,^19,20^ and encapsulation efficiencies are low for molecules with limited hydrophobicity (Log *P* < 5).^21–23^

**Figure 1:**
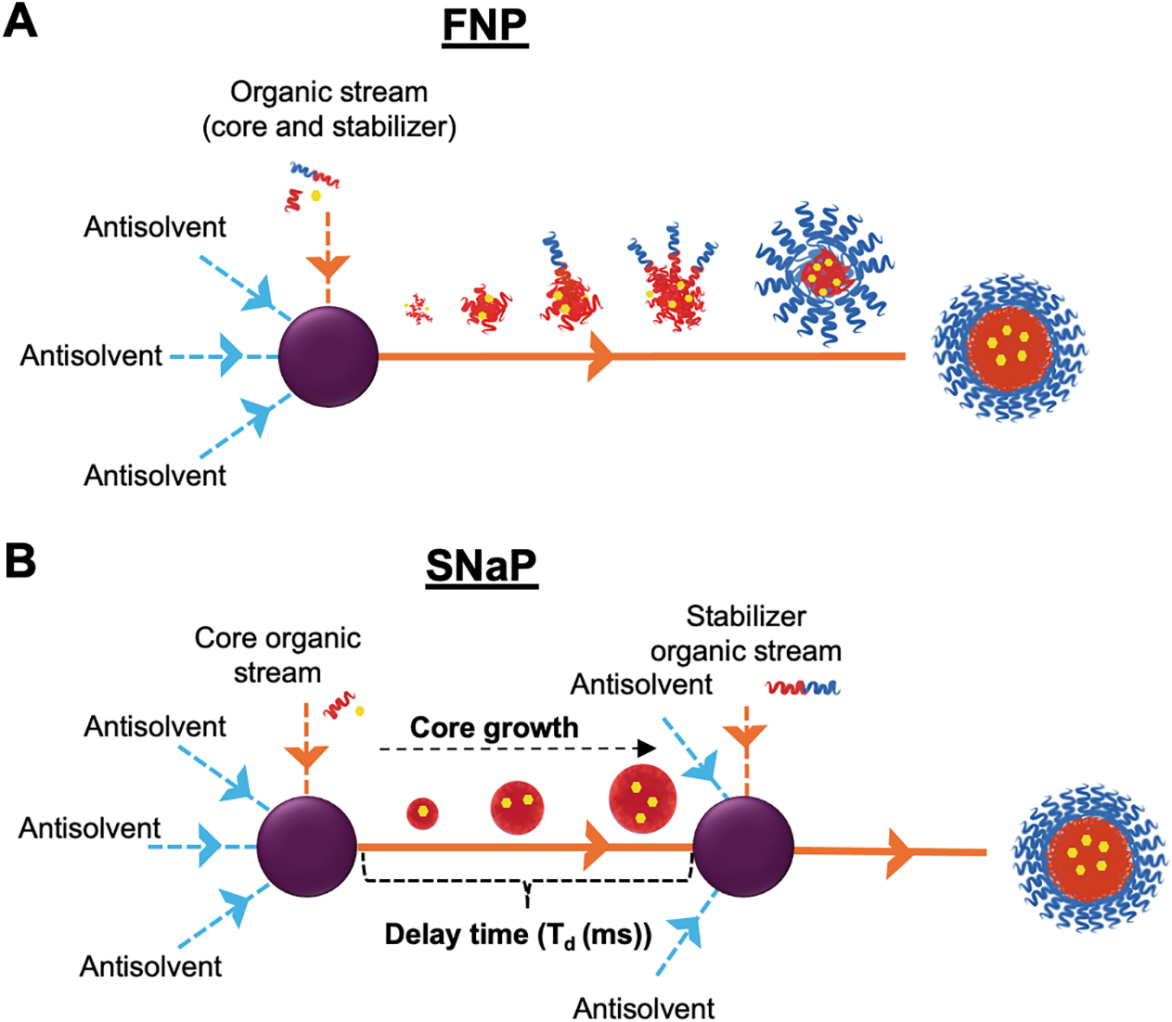
(A) Schematic of FNP setup. (B) Schematic of SNaP setup.

Sequential NanoPrecipitation (SNaP) is an emerging nanoparticle synthesis process that builds on the self-assembly principles of FNP. In SNaP, the particle core formation and stabilization are decoupled and controlled via the sequential addition of components (Figure 1B).^19,24^ To achieve sequential reagent addition with millisecond time control required for nanoparticle formation, two MIVM micromixers are connected in series.^19,25^ In the first mixing step, an organic stream containing the dissolved core components is rapidly mixed against antisolvent aqueous streams, which initiates the core nucleation and growth. The output from this mixer flows into the second mixer, where it is mixed against an organic stream containing dissolved amphiphilic BCP and antisolvent aqueous streams. The BCP stabilizer added in the second mixing step arrests the core growth and ensures the stability of the formed nanoparticles. We previously demonstrated that the delay time between core formation and particle stabilization steps is a tunable lever for the precise control of nanoparticle size.^26^ By tuning the delay time, a larger range of particle sizes, from nanometers to microns, can be synthesized via SNaP.^26,27^ Moreover, we have shown an improvement in the loading uniformity of inorganic components into nanoparticles with SNaP compared to FNP.^19^ These findings suggest SNaP overcomes two of the three FNP limitations. However, the encapsulation via SNaP of molecules of varying hydrophobicities has not been explored. Thus, it is unknown if SNaP can overcome the third FNP limitation.

In this study, we systematically compare polymeric nanoparticle formation using FNP and SNaP to understand how the synthesis process impacts particle properties. We examine how the solids concentration affects nanoparticle size and, importantly, evaluate how drug hydrophobicity impacts encapsulation efficiency across compounds ranging from highly hydrophobic β-carotene to moderately water-soluble ibuprofen. Additionally, we investigate differences in drug release profiles using itraconazole as a model compound. Our findings demonstrate that while FNP remains effective for nanoformulation of highly hydrophobic drugs, SNaP offers significant advantages in size tunability, improved encapsulation for moderately hydrophobic compounds, and enhanced control over drug release kinetics.

## 2 Materials and Methods

### 2.1 Materials

Tetrahydrofuran (THF, HPLC grade), acetonitrile (ACN, HPLC grade), and cinnarizine (98%) were purchased from Fisher Scientific. Ibuprofen, β-carotene were purchased from Sigma–Aldrich. 2-Hydroxy-1-napthaldehyde (xinafoic acid) and rubrene were purchased from Thermo Fisher Scientific. Poly­(d, l-lactide) (PLA, 10–18 kDa) and poly­(lactic acid)–poly­(ethylene glycol) (PEG–PLA, 5k-5k) were purchased from Evonik Inc. Ultrapure water (18.2 MΩ·cm) was generated from a MilliporeSigma Milli-Q Water Purification System.

For the three-dimensional (3D) printed mixers assembly, Tefzel tubing (inlet ID = 0.040″; outlet ID = 1/16″), female Luer tight syringe fitting systems (1/16″ OD), VacuTight Fittings (1/4–28–1/8), and flangeless male nut fittings (1/4–28, 1/16″) were purchased from Idex Health and Science. O-rings (75 Viton, 1.5 mm × 35 mm) and heated inserts for inlets were purchased from McMaster-Carr. The stainless-steel multi-inlet vortex mixer (MIVM) was purchased from Holland Applied Technologies.

### 2.2 Methods

#### 2.2.1 Nanoparticle Formation via Sequential Nanoprecipitation

NPs were synthesized via SNaP using a 3D-printed 4–4 mixer configuration as previously described.^26^ Prior to and following each synthesis, all mixer streams were flushed with 3 mL of tetrahydrofuran (THF) followed by 3 mL of ultrapure water. Flow rates were precisely controlled using a PHD Ultra Harvard Apparatus syringe pump.^28^ To ensure representative samples characteristic of continuous flow operation, collection began only after the flow had fully developed.^26,27^ This was achieved by discarding the first ∼7 mL of the process volume. The resulting solution was collected in a 4 °C Milli-Q water quenching bath to reduce the final organic concentration to less than 10 vol % THF.

For solids concentration studies, we maintained a constant core (PLA) to stabilizer (PEG–PLA) ratio of 1 to 2 by weight while varying the total solids concentration from 5 to 60 mg/mL. The synthesis process involved two sequential mixing stages. In the first stage, a THF stream containing dissolved PLA was mixed against three streams of ultrapure water. The output from this stage then entered the second mixing stage, where it was mixed against one THF stream containing dissolved PEG–PLA and two additional streams of ultrapure water. Each stream was maintained at a flow rate of 30 mL/min, establishing a fixed intermixer delay time of 7.8 ms.

For drug-loaded nanoparticle formulations, we employed a consistent two-stage mixing protocol with variations only in the composition of the first organic stream. The first THF stream contained the target drug and dissolved PLA at specific concentrations: cinnarizine (1.5 mg/mL) with xinafoic acid as a hydrophobic counterion (1.53 mg/mL) and PLA (6.97 mg/mL); ibuprofen (4.5 mg/mL) and PLA (5.5 mg/mL); β-carotene (6 mg/mL) and PLA (4 mg/mL); or itraconazole (3 mg/mL) and PLA (7 mg/mL). PLA was added as coexcipient to match the FNP formulations, which used PLA as coexcipient to provide better control over drug release kinetics.^29^ In all cases, this organic stream was mixed against three ultrapure water streams in the first stage. The output then entered the second mixing stage, where it combined with a THF stream containing dissolved PEG–PLA (20 mg/mL) and two additional ultrapure water streams. For all drug-loaded formulations, each stream flow rate was set to 20 mL/min. At these flow rates, the delay time was 10 ms.

All nanoparticle formulations underwent purification via dialysis using regenerated cellulose tubing (12–14 kDa MWCO, Repligen) against a 1:100 volume ratio of sample to ultrapure water for 6 h with hourly water changes. For ibuprofen-loaded nanoparticles, the dialysis protocol was modified to include water changes every hour for the first 5 h, followed by an overnight final equilibration.

#### 2.2.2 Nanoparticle Formation via Flash Nanoprecipitation

NPs were synthesized via FNP using the stainless-steel multi-inlet vortex mixer (MIVM). Before and following the NP synthesis, all inlets were first flushed with 3 mL of THF, followed by 3 mL of ultrapure water. Mixing was achieved by manual depression of the four syringes connected to the MIVM inlets evenly and synchronously as described by Markwalter et al.^5^ The resulting solutions were collected in a 4 °C Milli-Q water quenching bath to reduce the final organic concentration to less than 10 vol % THF.

For solids concentration studies, we maintained a constant core (PLA) to stabilizer (PEG–PLA) ratio of 1 to 2 by weight while varying the total solids concentration from 5 to 60 mg/mL. A THF stream containing dissolved PLA and PEG–PLA was prepared. This organic stream was mixed against three streams of ultrapure water. This solution was collected in a 4 °C MillQ water quenching bath to reduce the final organic concentration to less than 10 vol % THF.

For drug-loaded nanoparticle formulations, we employed a consistent FNP protocol with variations only in the composition of the organic stream. The THF stream contained the target drug, PLA, and PEG–PLA at specific concentrations: β-carotene (6 mg/mL), PLA (4 mg/mL), and PEG–PLA (20 mg/mL); cinnarizine (1.5 mg/mL) with xinafoic acid as a hydrophobic counterion (1.53 mg/mL), PLA (6.97 mg/mL), and PEG–PLA (20 mg/mL); itraconazole (3 mg/mL), PLA (7 mg/mL), and PEG–PLA (20 mg/mL); or ibuprofen (4.5 mg/mL), PLA (5.5 mg/mL), and PEG–PLA (20 mg/mL). PLA was added as coexcipient as is routinely done in the literature to provide better control over drug release kinetics.^29^ In all cases, this organic stream was mixed against three ultrapure water streams in a single mixing step. To ensure robustness of mixing by manual depression, the β-carotene experiments were also replicated using syringe pumps with each stream at a flow rate of 35 mL/min. The results can be found in the SI (Figure S5).

NPs were purified via dialysis to remove unencapsulated drug, counterions, and any remaining organic solvent. Using regenerated cellulose dialysis tubing (12–14 kDa MWCO, Repligen), particles were dialyzed against a 1:100 volume ratio of sample to ultrapure water for 6 h with hourly water changes. For ibuprofen-loaded nanoparticles, the dialysis protocol was modified to include water changes every hour for the first 5 h followed by an overnight final equilibration.

#### 2.2.3 Itraconazole Release Assay

For the itraconazole release assay, the nanoparticle dispersion was diluted using a solution of 10× PBS and 20% Tween to achieve a final concentration of 1× PBS and 2% Tween. One mL of this solution was placed in a dialysis bag that was immersed in a vial containing 10 mL of release medium that consists of 1× PBS and 2% Tween. This vial was then placed in a water bath maintained at a temperature of 37 °C under mild shaking. At each time point, 250 μL of the release medium was collected to be later analyzed using HPLC.

#### 2.2.4 Nanoparticle Characterization

Nanoparticle size was measured via dynamic light scattering (DLS) using a ZetaSizer Nano ZS (Malvern Instruments) using a detection angle of 173° at 25 °C. NP samples were diluted 10× in ultrapure water. The intensity-average diameter as presented by the Malvern deconvolution software, general purpose mode, is reported, with standard deviation reported across three measurements.

Total particle solids concentration was determined using a thermogravimetric analyzer (TGA, Discovery 550 TGA, TA Instruments) as previously described.^26^

β-Carotene concentration was determined using the Duetta spectrophotometer-fluorimeter (Horiba) by measuring the absorbance spectra at 460 nm (see Supporting Information (SI) Figure S1). The sample was prepared by diluting the NP solution in THF to achieve a final 10:90 vol/vol water to THF ratio.

Concentration of cinnarizine, itraconazole, and ibuprofen was determined via high-performance liquid chromatography on a ThermoFisher Vanquish Core HPLC equipped with an autosampler, quaternary pump, UV detector, and column (Hypersil Gold C18, 3 μm particle size, 175 Å pore size, 4.6 mm × 150 mm, 30 °C). Details of the calibration curves are presented in the Supporting Information (Figures S2–S4).

Nanoparticle drug loading (DL), which is the percent of drug by mass in nanoparticles, was determined using the equation

$$\mathrm{DL}=\frac{C_{\mathrm{drug}}}{C_{\mathrm{total}}}\times 100$$

where *C*_drug_ is the concentration of drug determined via HPLC and *C*_total_ is the total solids concentration (polymer and drug) determined via TGA.

Encapsulation efficiency (EE%), which is the percent of drug encapsulated into the nanoparticle relative to the amount of drug engaged into the process, was determined using the equation

$$\mathrm{EE}=\frac{\mathrm{DL}}{{\mathrm{DL}}_{\mathrm{target}}}\times 100$$

The DL_target_ is the attempted drug loading for each formulation.

For cinnarizine, HPLC samples were prepared by diluting NP solutions into acetonitrile to achieve a final 40:60 vol/vol water to acetonitrile ratio. The following isocratic mobile phase was used: 60 vol % acetonitrile with 0.1 vol % trifluoracetic acid and 40 vol % water with 0.1 vol % trifluoracetic acid, with a total flow rate of 0.5 mL/min. Detection was performed at 224 nm.

For ibuprofen, HPLC samples were prepared by diluting NP solutions into acetonitrile to a final 30:70 vol/vol water to acetonitrile ratio. The following isocratic mobile phase was used: 70 vol % acetonitrile with 0.1 vol % trifluoracetic acid and 30 vol % water with 0.1 vol % trifluoracetic acid, with a total flow rate of 0.5 mL/min. Detection was performed at 224 nm.

For itraconazole, HPLC samples were prepared by diluting NP solutions into acetonitrile to a final 50:50 vol/vol water and acetonitrile ratio. The mobile phase was 50 vol % acetonitrile with 0.1 vol % trifluoracetic acid and 50 vol % water with 0.1 vol % trifluoracetic acid, with a total flow rate of 0.5 mL/min. Detection was performed at 256 nm.

Nanoparticles were visualized via transmission electron microscopy (TEM) using a FEI Talos L120C microscope. To prepare the TEM sample, a 0.5 mg mL–1 NP solution was deposited onto the Formvar on carbon, 300 mesh Cu TEM grid (TedPella) and allowed to dry. Negative staining was done with 2% phosphotungstic acid (PTA), pH 6. The TEM imaging was performed using the instrument and facilities at NYU Langone Microscopy Laboratory (RRID: SCR_017934)

## 3 Results and Discussion

Herein, we systematically compare the formation of nanoparticles with PLA cores and PEG stabilizing coronas synthesized via FNP and SNaP under identical conditions (i.e., components, component concentrations, and solvent system). First, we investigate how varying total component concentration affects nanoparticle size control for both techniques. Next, we assess encapsulation efficiency across a spectrum of drug hydrophobicity, examining compounds ranging from highly hydrophobic β-carotene (Log *P* 13.5) to moderately water-soluble ibuprofen (Log *P* 3.5). Finally, we characterize and compare drug release kinetics using itraconazole as a model compound, providing insights into how the distinct assembly processes influence the drug release rate and particle stability.

### 3.1 Influence of the Solids Concentration on Nanoparticle Size

The total solids concentration (i.e., the total component concentration) is a key parameter for controlling polymeric nanoparticle size in rapid precipitation techniques, as it determines the degree of core material supersaturation, which directly influences the particle core nucleation and growth kinetics.^30^ We investigated how varying the total solids concentration of the PLA core and PLA–PEG BCP stabilizer, while maintaining a constant core-to-stabilizer mass ratio, affected particle size in both FNP and SNaP. All the other parameters were kept constant between the two synthesis methods, with SNaP utilizing a fixed delay time of 7.8 ms.

For FNP, nanoparticle assembly was not achieved at the lowest concentration tested (5 mg/mL), as evidenced by multiple populations in the DLS trace (Figure 2A). This indicates that in these dilute conditions, the supersaturation required for stable nanoparticle formation was not attained; The assembly of the core components was too slow relative to the micellization of the BCP stabilizer. However, as the concentration increased, stable nanoparticles with narrower polydispersities formed with sizes increasing from 35 ± 4 nm (PDI = 0.4 ± 0.12) at 10 mg/mL to 75 ± 1 nm (PDI = 0.15 ± 0.01) at 60 mg/mL (Figure 2A,C), demonstrating concentration-dependent growth behavior, consistent with previous literature.^7,18^

**Figure 2:**
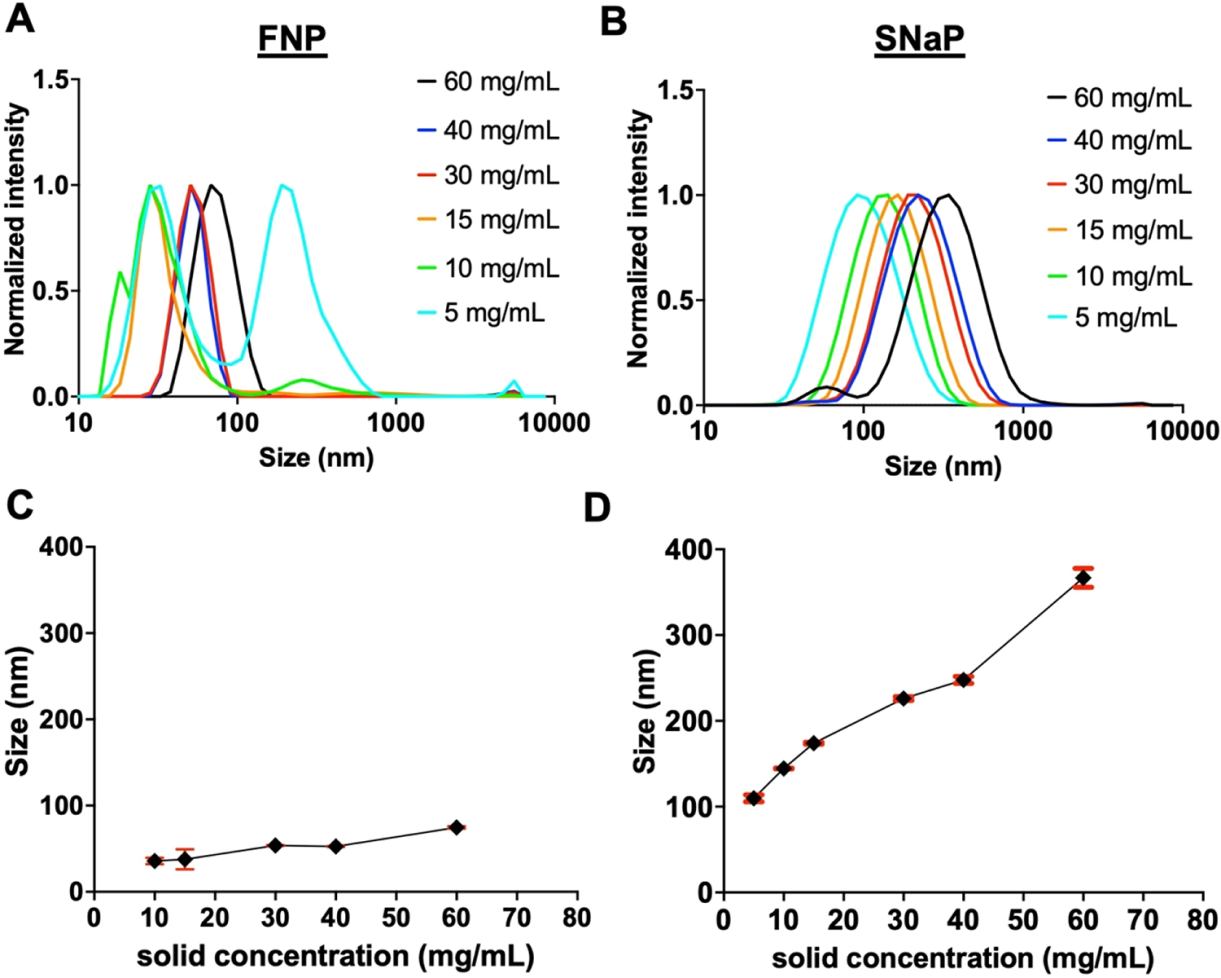
Influence of solid concentration on PEG–PLA nanoparticles formed through FNP and SNaP. (A) Normalized intensity weighted DLS traces of PEG–PLA nanoparticles formed via FNP with varying solid concentrations. (B) Normalized intensity weighted DLS traces of PEG–PLA nanoparticles formed via SNaP with varying solid concentrations. (C) Size variation in function of solid concentration for nanoparticles formed through FNP (*N* = 3). (D) Size variation in function of solid concentration for nanoparticles formed through SNaP (*N* = 3).

In contrast, SNaP successfully produced stable nanoparticles with narrow polydispersities even at 5 mg/mL, suggesting that delaying stabilization by a few milliseconds allowed sufficient time for the nanoparticle core to form prior to stabilization (Figure 2B). This is particularly advantageous for the formulation of costly small-molecule therapeutics, where only minimal quantities of the valuable active pharmaceutical ingredient are accessible. As with FNP, increasing solid concentration resulted in progressively larger particles, with sizes ranging from 109 ± 4 nm (PDI = 0.18 ± 0.01) at 5 mg/mL to approximately 367 ± 11 nm (PDI = 0.27 ± 0.01) at 60 mg/mL (Figure 2B,D). Complete formulation and size data for FNP and SNaP nanoparticles are tabulated in Table 1. These results demonstrate that while both methods exhibit concentration-dependent growth, SNaP enables nanoparticle formation at lower solid concentrations and provides access to a significantly broader size range.

**Table 1:** Average Size and PDI of PEG–PLA Nanoparticles Formed through FNP and SNaP

	total solid concentration (mg/mL)	peak 1 diameter (nm)	peak 2 diameter (nm)	PDI
FNP	5	NA	NA	NA
	10	35 ± 4	175 ± 144	0.4 ± 0.12
	15	38 ± 11	579 ± 516	0.34 ± 0.11
	30	53 ± 1	NA	0.2 ± 0.02
	40	53 ± 1	NA	0.19 ± 0.04
	60	75 ± 1	NA	0.15 ± 0.02
SNaP	5	109 ± 4	NA	0.18 ± 0.01
	10	144 ± 1	NA	0.16 ± 0.01
	15	174 ± 1	NA	0.16 ± 0.01
	30	226 ± 2	NA	0.18 ± 0.01
	40	247 ± 4	NA	0.21 ± 0.01
	60	367 ± 11	62 ± 3	0.27 ± 0.01

This difference in size sensitivity to the total solids concentration can be attributed to fundamental differences in nanoparticle assembly mechanisms between the two processes. In FNP, the core formation and stabilization by the BCP occur simultaneously (Figure 1A). The total assembly time, and consequently the particle size, depends on the ratio of core material to BCP.^18^ The more BCP stabilizer, the faster the nanoparticle growth is arrested, resulting in smaller nanoparticles.^18^ To account for the BCP participation, Pagels and Prud’homme developed a scaling law for FNP based on Smoluchowski’s model of diffusion-limited growth (eq 1)­

1 $$R={\left(K\frac{k_{B}TC_{\mathrm{core}}^{5/3}}{\pi \mu \rho C_{\mathrm{BCP}}}\right)}^{1/3}$$

where *R* is the nanoparticle radius, *K*_B_ is Boltzmann’s constant, *T* is temperature, μ is the solvent viscosity, ρ is the density, *C*_core_ is the core concentration, *C*_BCP_ is the stabilizer concentration, and *K* is a system-dependent constant.^18^ Notably, this equation contains no explicit time dependence. The particle radius depends on the core-to-stabilizer ratio.

SNaP, however, decouples core formation from stabilization, allowing the core to assemble and grow until stabilizer addition in the second mixing step. Therefore, particle size becomes independent of BCP concentration, provided sufficient stabilizer is present to rapidly coat the formed cores. Therefore, SNaP particle assembly can be described by Smoluchowski’s model of diffusion-limited growth (eq 2)­

2 $$R={\left(\frac{tk_{B}Tc_{\mathrm{core}}}{\pi {\mu \rho }_{\mathrm{core}}}\right)}^{1/3}$$

where *R* is the nanoparticle radius, *t* is the assembly time (equivalent to the intermixer delay time), *K*_B_ is Boltzman’s constant, *T* is temperature, μ is the solvent viscosity, ρ is the density, and *C*_core_ is the core concentration.^26^ From the equation, we see that the nanoparticle size depends on the core concentration and the delay time, two levers that we can control experimentally. We previously demonstrated that particle growth scales with delay time in alignment with this model. Here, we show that particle size also depends on total solids concentration.

To compare the concentration dependence between the two processes, we can simplify the Pagels and Prud’homme scaling law by assuming that *C*_core_ is approximately equal to *C*_BCP_. This is reasonable given that the ratio of *C*_core_ to *C*_BCP_ generally ranges from 1:3 to 3:1 in FNP formulations, therefore, the concentrations are the same order of magnitude. With this assumption, eq 1 simplifies to

3 $$R={\left(K\frac{k_{B}TC_{\mathrm{core}}^{2/3}}{\pi \mu \rho }\right)}^{1/3}$$

Now the nanoparticle size only depends on *C*_core_, enabling us to compare directly with the Smoluchowski model. We see that in FNP, nanoparticle size scales with *C*_core_ to the 2/9 power, while in SNaP, nanoparticle size scales with *C*_core_ to the 1/3 power. The stronger concentration dependence in SNaP explains the broader size range observed in our experiments and establishes total solids concentration as a more powerful formulation lever for size tuning in SNaP compared to FNP. However, this increased sensitivity to stream concentration represents a potential liability, thereby necessitating robust control over component concentrations.

### 3.2 Impact of Drug Hydrophobicity on Encapsulation Efficiency

To compare how drug hydrophobicity influences encapsulation efficiency in FNP and SNaP, we evaluated the formation of drug-loaded PEG–PLA nanoparticles across compounds with varying Log *P* values. In precipitation-driven assembly methods, the drug supersaturation is critically important as it controls the nucleation rate and growth rate.^31^ The supersaturation in the rapid solvent-switching process is commonly expressed as shown in eq 4

4 $$S=\frac{C}{C_{\mathrm{eq}}}$$

where *S* is the supersaturation, *C* is the drug solution concentration and *C*_eq_ is the equilibrium drug concentration.^32^ For precipitation in aqueous antisolvents, *C*_eq_ varies inversely with Log *P*; thus, it is easier to achieve high supersaturation for drugs with high Log *P* values.

Given FNP’s fundamental reliance on high supersaturation to achieve rapid nucleation and core growth prior to BCP stabilization, we anticipated high encapsulation efficiency for highly hydrophobic compounds (Log *P* > 5). While for weakly hydrophobic compounds (3 > Log *P* > 5) with lower supersaturation and slower nucleation and growth relative to BPC assembly, we anticipated low encapsulation efficiencies in FNP nanoparticles, consistent with previous literature.

In contrast, SNaP decouples core formation from stabilization, potentially making the process less dependent on achieving high supersaturations due to the delayed addition of the BCP stabilizer. In SNaP, weakly hydrophobic drugs have more time to nucleate and assemble into the core prior to the introduction of stabilizer relative to FNP, making the process less dependent on achieving high supersaturation and thus less sensitive to drug Log *P*. This reduced dependence on high supersaturation could result in a broader range of drugs that can be successfully encapsulated via SNaP compared to FNP. This comparative analysis across the hydrophobicity spectrum enabled us to establish clear relationships between a drug’s Log *P* value and its encapsulation behavior in each nanoprecipitation method.

#### 3.2.1 β-Carotene Encapsulation

We began our studies with the extremely hydrophobic, β-carotene, which has an octanol–water partition coefficient (log *P*) of 13.5 (Figure 3A). β-carotene is a hydrophobic food-derived pigment that can be converted in the body into vitamin A.^33^ Due to its fat-soluble nature, its bioavailability is limited.^34^ Consequently, various strategies have been developed to enhance its absorption, including encapsulation in nanoparticles, which has been successfully achieved via FNP.^6,35,36^ For the comparison between FNP and SNaP, we used a formulation with a target drug loading of 20 wt %.

**Figure 3:**
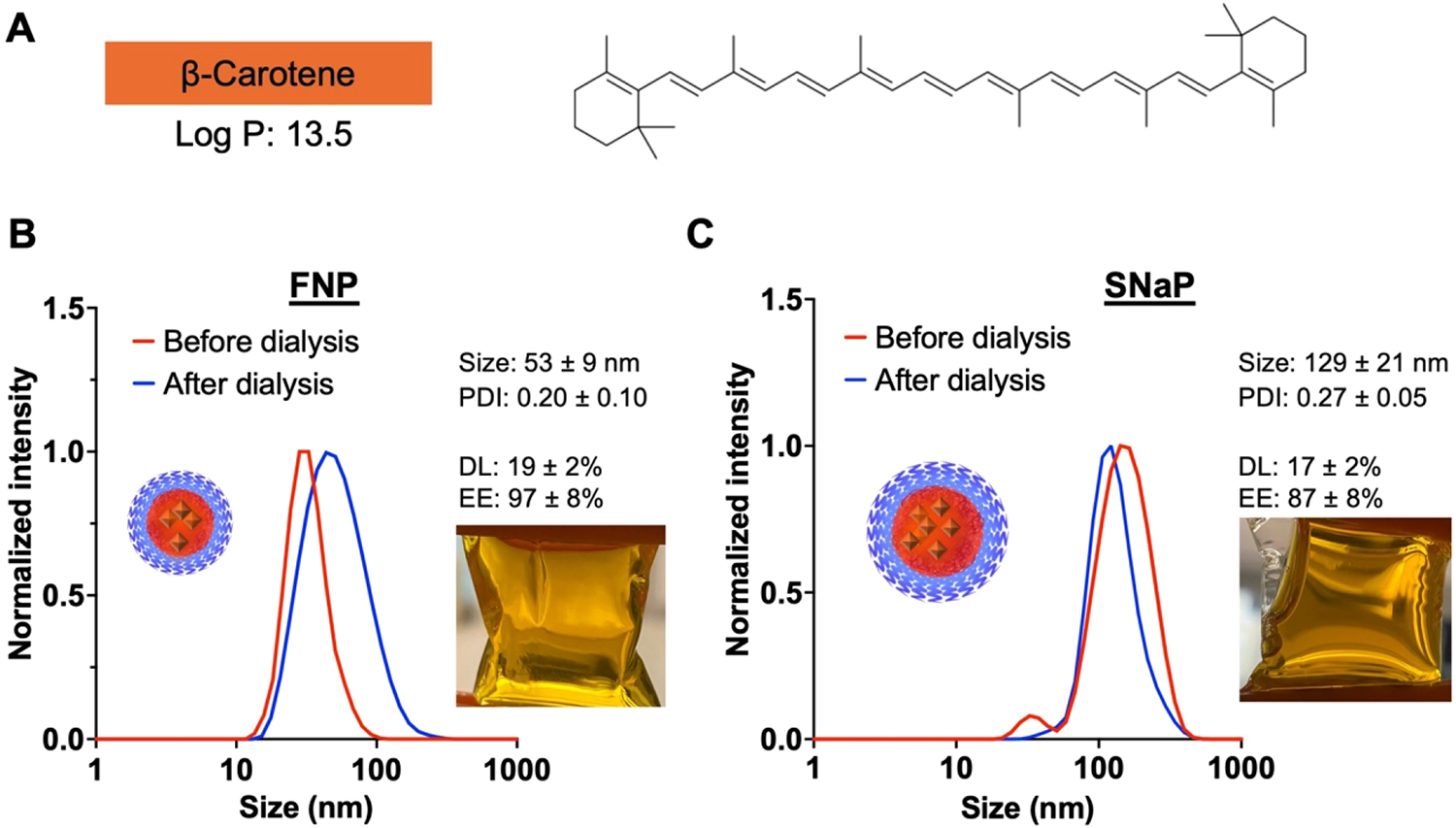
β-Carotene encapsulation in PEG–PLA nanoparticles formed via FNP and SNaP. (A) β-carotene molecular structure. (B) Normalized intensity weighted DLS trace of β-carotene loaded PEG–PLA nanoparticles formed via FNP before and after dialysis (*N* = 3). The photo shows the β-carotene-loaded PEG–PLA nanoparticle formed via FNP after dialysis. (C) Normalized intensity weighted DLS trace of β-carotene loaded PEG–PLA nanoparticles formed via SNaP before and after dialysis (*N* = 3). The photo shows the β-carotene-loaded PEG–PLA nanoparticle formed via SNaP after dialysis.

β-Carotene was successfully encapsulated using both FNP and SNaP methods. Due to its high hydrophobicity, no precipitate was observed in either case after dialysis (Figure 3B,C insets), indicating efficient encapsulation. The encapsulation efficiency achieved with FNP was 97 ± 8%, consistent with previous literature,^36,37^ compared to 87 ± 8% for SNaP. Despite their similar high encapsulation efficiencies, each process produced nanoparticles of different sizes. FNP produced smaller particles with diameters of 53 ± 9 nm (PDI = 0.20 ± 0.1) (Figure 3B) compared to SNaP, which produced larger particles with diameters of 129 ± 21 nm (PDI = 0.27 ± 0.05) (Figure 3C). This size difference can be attributed to the delayed stabilization in SNaP, which allows continued core growth before stabilizer addition arrests further aggregation. For highly hydrophobic compounds like β-carotene, both methods achieve comparable encapsulation efficiency, suggesting that the choice between methods may depend primarily on the desired particle size rather than drug loading considerations.

#### 3.2.2 Cinnarizine Encapsulation

We next investigated the encapsulation of cinnarizine, an antihistamine, which is weakly hydrophobic with a log *P* of 5.7 (Figure 4A).^38^ Due to its low hydrophobicity, previous studies have shown that the hydrophobic ion pairing (HIP) solubility engineering approach is required to encapsulate cinnarizine into nanoparticles via FNP.^39^ In the HIP nanoformulation approach, an ionizable hydrophobic counterion forms a hydrophobic salt with the ionizable drug, thereby enhancing its hydrophobicity and promoting nucleation and particle growth during FNP.^40^ We used xinafoic acid (Log *P* 3.2) as the counterion to form an HIP between cinnarizine’s protonatable tertiary amine and xinafoic acid’s carboxylic acid group (Figure 4A,B). For the comparison between FNP and SNaP, we used a formulation with a target drug loading of 5 wt %.

**Figure 4:**
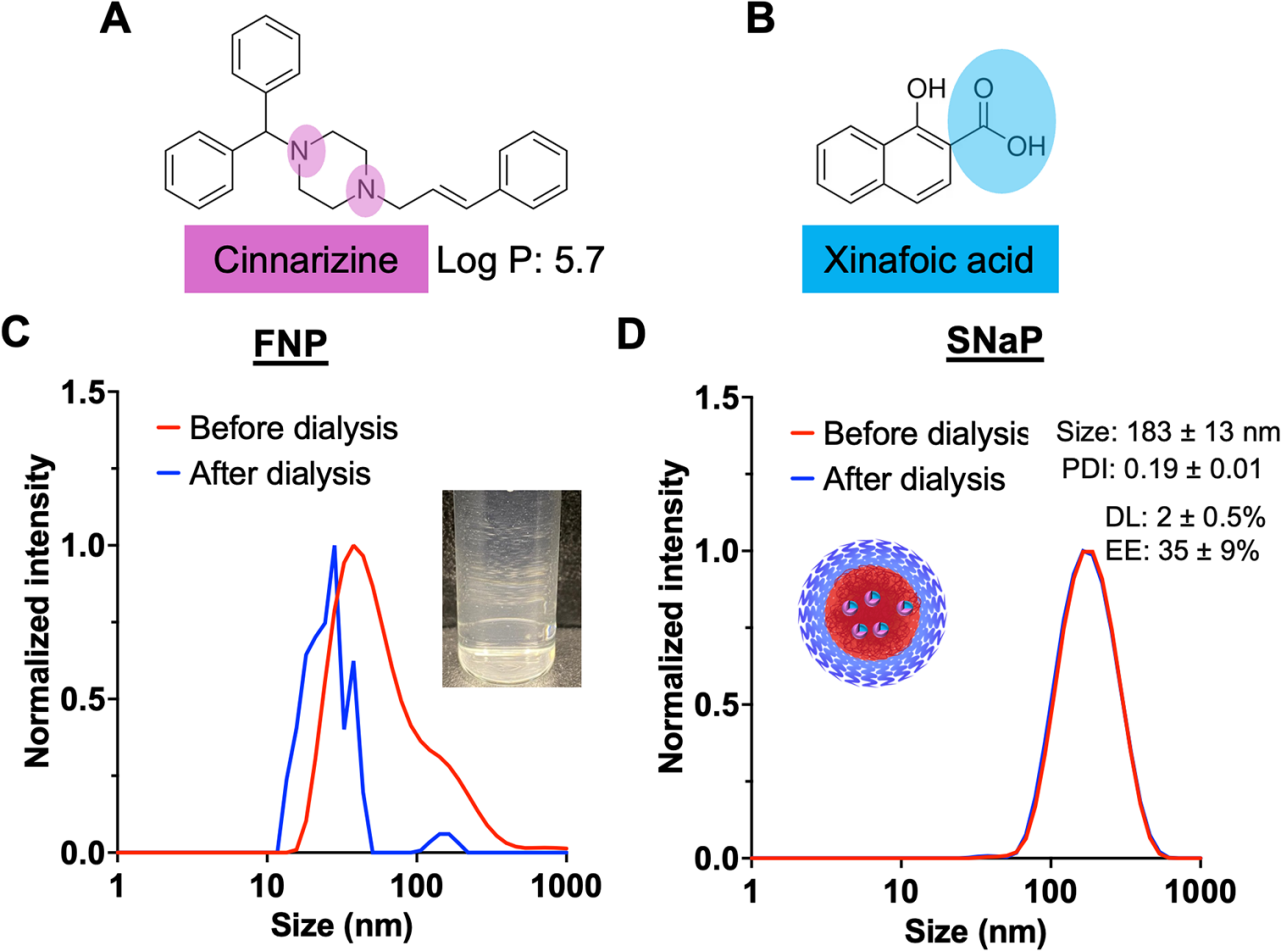
Cinnarizine encapsulation in PEG–PLA nanoparticles formed via FNP and SNaP. (A, B) Molecular structures of cinnarizine (A) and xinafoic acid (B) with functional groups involved in HIP highlighted: The protonatable tertiary amine in cinnarizine and the carboxylic acid group in xinafoic acid (C). Normalized intensity weighted DLS traces of cinnarizine-loaded PEG–PLA nanoparticles formed via FNP before and after dialysis (*N* = 3). The photo shows the visible precipitate in the cinnarizine-loaded nanoparticles formed via FNP after dialysis (D). Normalized intensity weighted DLS trace of cinnarizine-loaded PEG–PLA nanoparticles formed via SNaP (*N* = 3) before and after dialysis. The photo shows the Cinnarizine-loaded PEG–PLA nanoparticle formed via SNaP after dialysis.

FNP formulations exhibited significant polydispersity and visible precipitate formation immediately after synthesis (Figure 4C). Following dialysis, substantial aggregation was observed, confirming the inability of FNP to form stable cinnarizine-loaded nanoparticles under these conditions. This can be attributed to either (1) insufficient time for effective hydrophobic ion pairing between cinnarizine and xinafoic acid to occur and drive rapid nucleation or (2) the HIP pair formed but was not sufficiently hydrophobic to achieve rapid nucleation during the single-step FNP process, preventing successful drug nucleation and core encapsulation.

In contrast, SNaP produced stable, monodisperse cinnarizine-loaded nanoparticles with diameters of 183 ± 13 nm (PDI = 0.19 ± 0.01) that remained intact after dialysis (Figure 4D). The final formulation achieved 2 ± 0.5 wt % drug loading with 35 ± 9% encapsulation efficiency. This marked improvement over FNP demonstrates the advantage of decoupling core formation and stabilization, providing critical additional time for hydrophobic ion pairing to occur and relaxing the requirement of high supersaturation and rapid nucleation, thereby enabling successful encapsulation of weakly hydrophobic drugs.

#### 3.2.3 Ibuprofen Encapsulation

Ibuprofen is an anti-inflammatory drug that is commonly used for pain management and fever reduction.^41^ With a Log *P* of 3.5 (Figure 5A), ibuprofen is only moderately hydrophobic, making its encapsulation into polymeric nanoparticles with hydrophobic cores particularly challenging, and consequently, only a few examples of such formulations have been reported in the literature.^42–44^ For the comparison between FNP and SNaP, we used a formulation with a target drug loading of 15 wt % ibuprofen.

**Figure 5:**
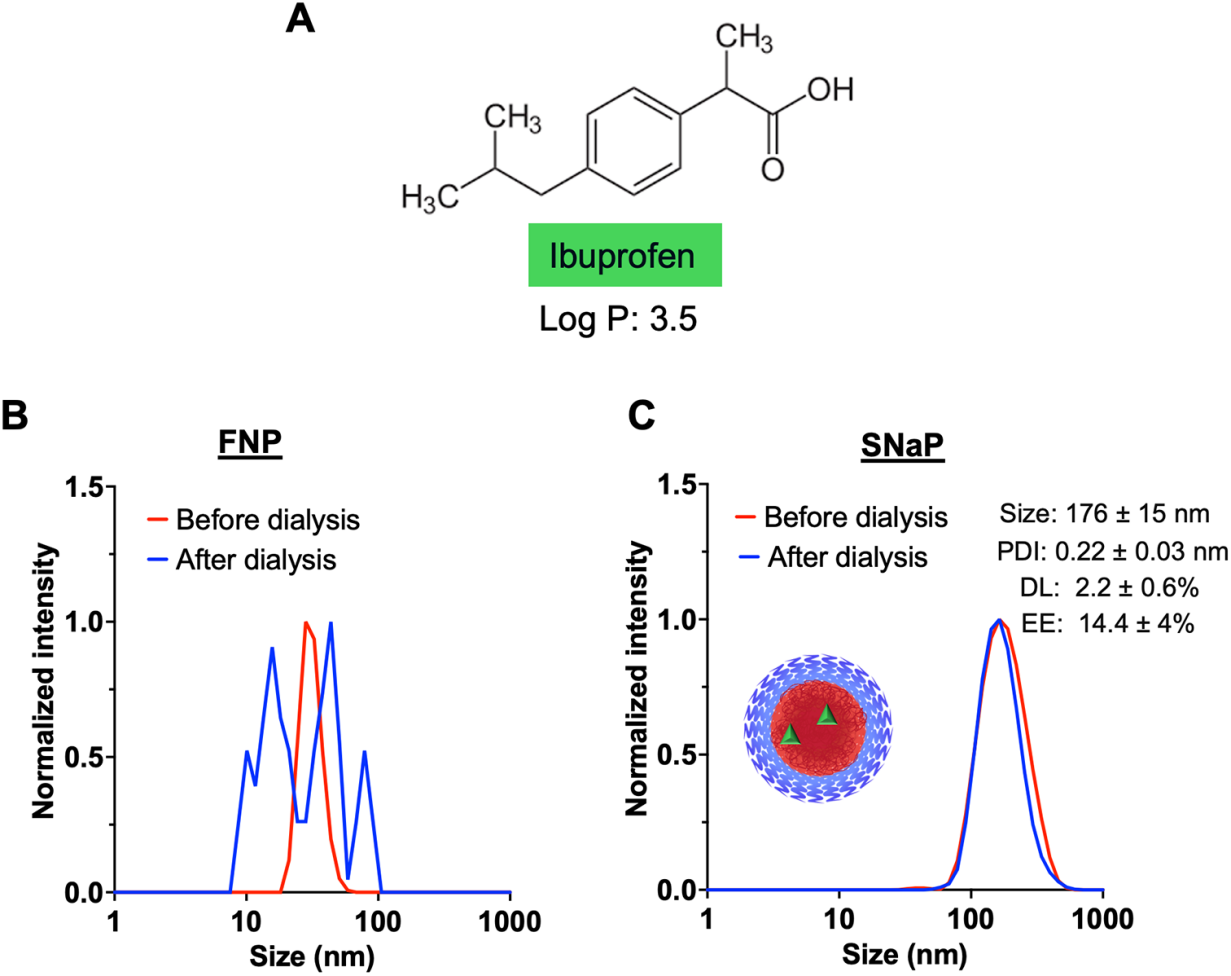
Ibuprofen encapsulation in PEG–PLA nanoparticles formed via FNP and SNaP. (A) ibuprofen molecular structure. (B) Normalized intensity weighted DLS trace of ibuprofen-loaded PEG–PLA nanoparticles formed via FNP before and after dialysis (*N* = 3). The photo shows the ibuprofen-loaded PEG–PLA nanoparticle formed via FNP after dialysis. (C) Normalized intensity weighted DLS trace of ibuprofen-loaded PEG–PLA nanoparticles formed via SNaP (*N* = 3). The photo shows the ibuprofen-loaded PEG–PLA nanoparticle formed via SNaP after dialysis.

Nanoparticles formed through FNP initially had a uniform size distribution, with an average diameter of 39 ± 2 nm (PDI = 0.15 ± 0.05) (Figure 5B, red trace). However, postdialysis, substantial aggregation was observed as evidenced by the change in the DLS trace after dialysis (Figure 6B, blue trace), indicating unstable encapsulation. The NP size post dialysis could not be determined. In contrast, SNaP produced ibuprofen-loaded nanoparticles that remained stable after dialysis (Figure 5C). The particle size was 176 ± 15 nm (PDI = 0.22 ± 0.03). The SNaP nanoparticles had a drug loading of 2.2 ± 0.6 wt % and an encapsulation efficiency of 14.4 ± 4%.

**Figure 6:**
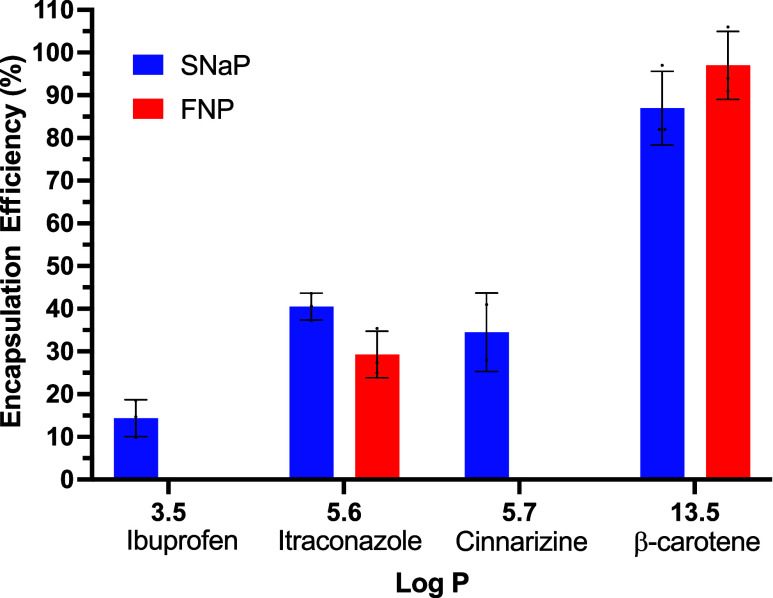
Encapsulation efficiency of small molecule therapeutics for FNP and SNaP processes as a function of molecule Log *P*. Bars without data (ibuprofen-FNP, cinnarizine-FNP) indicate no encapsulation.

While the encapsulation efficiency was low for SNaP nanoparticles, this represents a clear improvement over FNP nanoparticles, which were unable to encapsulate ibuprofen. This suggests that SNaP is more efficient at encapsulating poorly hydrophobic drugs compared to FNP. The improved performance of SNaP can be attributed to the delay time between core formation and stabilization, which allows additional time for drugs to nucleate and aggregate, making the process less dependent on achieving supersaturation compared to FNP. SNaP opens the possibility of encapsulating a broader range of therapeutic small molecules into nanoparticles.

#### 3.2.4 Correlation between Log *P* and Encapsulation Efficiency

When plotted together, our results confirm the strong relationship between drug hydrophobicity (Log *P*) and encapsulation efficiency for both methods (Figure 6, which for completeness, includes itraconazole data from Section 3.3). As Log *P* values increase, indicating greater hydrophobicity, encapsulation efficiency improves regardless of the method used. This trend can be attributed to the nucleation-based mechanism underlying both processes. Highly hydrophobic compounds achieve supersaturation upon mixing with the antisolvent, initiating rapid nucleation and growth.^45^ Hence, drugs with higher hydrophobicity exhibit low equilibrium solubility, which allows achieving high supersaturation levels even at low concentration. Therefore, the rapid precipitation facilitates efficient encapsulation within the forming nanoparticle core.^45^ This is exemplified by β-carotene, which we estimate has a supersaturation of 3840 in both processes and the highest encapsulation efficiency (See SI Table 1 for calculation details). Conversely, compounds with lower hydrophobicity exhibit slower nucleation rates and lower supersaturation, resulting in reduced encapsulation efficiency.^45^ This is exemplified by the remaining four compounds tested. We estimate a supersaturation of 190 for cinnarizine, 80 for itraconazole and only 20 for ibuprofen (See SI Table 1 for calculation details).

This effect is particularly pronounced in FNP, where core formation and stabilization occur simultaneously. The drug molecules must precipitate at rates comparable to or faster than the BCP assembly, which is on the order of 5–10 ms for PLA–PEG.^46–49^ The sequential approach of SNaP, by contrast, introduces a critical delay between core formation and stabilization. This temporal separation provides additional time for drug nucleation and growth before the addition of stabilizing polymers, enabling more complete drug incorporation. This is similar to the batch, in situ sequential precipitation achieved by Liu et al., who used complex solvent systems to initiate small molecule precipitation followed by polymer assembly.^50^ Consequently, SNaP consistently achieves higher encapsulation efficiencies for moderately hydrophobic compounds compared to conventional FNP.

### 3.3 Comparison of Itraconazole-Loaded Nanoparticles Synthesized via FNP and SNaP

Although hydrophobicity drives self-assembly in both FNP and SNaP, the fundamental assembly mechanisms differ significantly. In FNP, core components and the stabilizing block copolymer assemble simultaneously, allowing the hydrophobic block of the BCP to directly influence core organization during formation. In contrast, SNaP first forms the hydrophobic core without BCP interference, followed by stabilizer addition in a separate step. This temporal separation likely creates differences in internal nanoparticle structure and organization, potentially affecting drug release behavior.

To evaluate these differences in release kinetics, we selected itraconazole, an antifungal drug with a Log *P* of 5.6, as our model compound (Figure 7A).^51^ This selection was based on three key considerations: (1) itraconazole has been successfully encapsulated into nanoparticles via FNP in previous studies, providing a benchmark;^52^ (2) its moderate hydrophobicity suggested it would be effectively encapsulated by both methods while potentially revealing differences in their performance; and (3) prior research has demonstrated that itraconazole release from polymeric nanoparticles is highly sensitive to core properties,^53^ making it an ideal probe for detecting structural differences resulting from the distinct assembly mechanisms.

**Figure 7:**
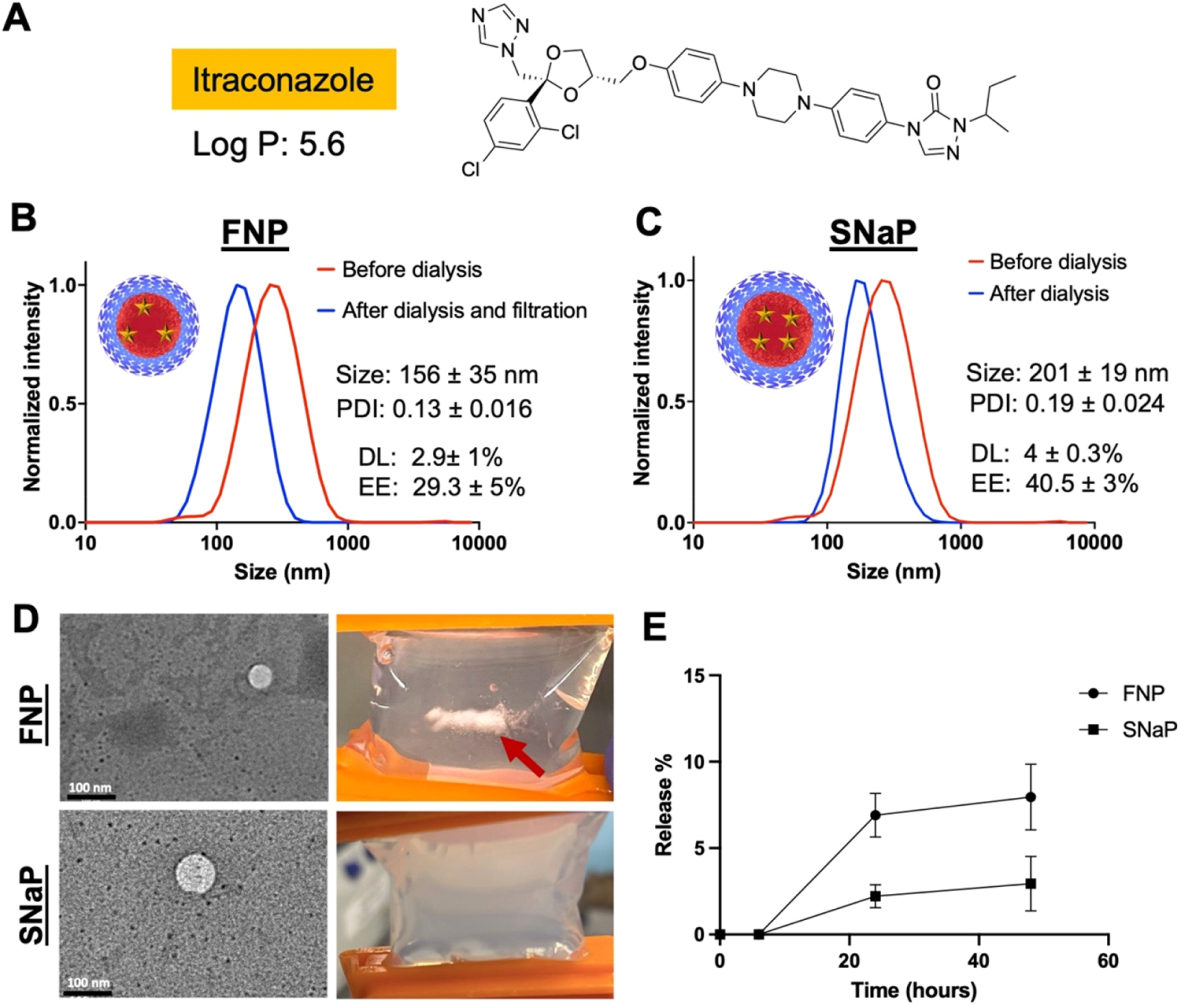
Itraconazole encapsulation in PEG–PLA nanoparticles formed via FNP and SNaP and their release assay. (A) Itraconazole molecular structure. (B) Normalized intensity weighted DLS trace of Itraconazole-loaded PEG–PLA nanoparticles formed via FNP (*N* = 2). (C) Normalized intensity weighted DLS trace of Itraconazole-loaded PEG–PLA nanoparticles formed via SNaP (*N* = 2). (D) TEM images of itraconazole-loaded PEG–PLA nanoparticles formed via FNP and SNaP. Both methods yielded spherical particles. The photos show the visible precipitate in the itraconazole-loaded nanoparticles formed via FNP after dialysis (red arrow) and the absence of precipitate in the itraconazole-loaded nanoparticles formed via SNaP. (E) Drug-release profiles of itraconazole-loaded nanoparticles formed via FNP and SNaP over 48 h (in PBS, pH 7.4, 37 °C). Nanoparticles formed via SNaP exhibited a slower release compared to the nanoparticles formed via FNP.

We initially made nanoparticles with a target drug loading of 20 wt % itraconazole. However, only the SNaP nanoparticles were stable, limiting our ability to compare particle release rates. (SI Figures S6–S9). Therefore, instead, we selected a formulation with a target drug loading of 10 wt % itraconazole for the comparison. FNP-produced nanoparticles exhibited visible precipitate formation during dialysis, requiring filtration (Figure 7D, red arrow). Postfiltration, the resulting particles had an average size of 156 ± 35 nm (PDI = 0.13 ± 0.016) with 2.9 ± 1 wt % drug loading and 29.3 ± 5% encapsulation efficiency (Figure 7B). SNaP-produced nanoparticles were completely stable with no visible precipitate (Figure 7C), with an average size of 200 ± 19 nm (PDI = 0.19), 4 ± 0.3 wt % drug loading, and 41 ± 3% encapsulation efficiency (Figure 7C). The SNaP process achieved a higher encapsulation efficiency compared to FNP, consistent with the cinnarizine and ibuprofen results. TEM imaging confirmed the formation of spherical nanoparticles by both methods (Figure 7D).

Release studies revealed significant differences in release kinetics. Initially, neither formulation showed detectable release. After 24 h, SNaP-produced nanoparticles released approximately 2.2 ± 0.6% of the encapsulated itraconazole with minimal further increase at 48 h. In contrast, FNP-produced nanoparticles released approximately 7 ± 1.3% after 24 h, increasing to 9 ± 1.9% after 48 h (Figure 7E). While both particles exhibited slow-release kinetics, there was a significant difference in their release rates, suggesting a difference in particle organization. The faster release observed for the FNP nanoparticles is likely attributable to incomplete incorporation of the drug into the hydrophobic core, with a fraction instead adsorbed to the core surface or residing within the PEG corona. While for SNaP nanoparticles, the delay in BCP addition could favor core encapsulation and minimize drug interaction with the PEG corona. These architectural distinctions at the molecular level may contribute to SNaP’s enhanced control over release kinetics despite using identical materials. Further studies are necessary to fully elucidate the differences in nanoparticle core organization.

### 3.4 FNP and SNaP Process Considerations

It is important to recognize that rapid nanoprecipitation methods such as FNP and SNaP present inherent limitations that may influence their applicability. A primary concern is the risk of oiling out and emulsion formation.^54^ In FNP, high supersaturation can promote liquid–liquid phase separation, resulting in kinetically trapped emulsions or uncontrolled particle growth rather than well-defined nanostructures.^55^ Because SNaP is less dependent on achieving high supersaturations, it may partially mitigate this limitation; however, the formation of emulsions remains a potential risk in both approaches if mixing and solvent conditions are not carefully optimized. In addition, both FNP and SNaP rely on high-Reynolds-number mixers to achieve rapid micromixing, which may adversely affect shear-sensitive drug–polymer assemblies.^56^ Finally, the solid form landscape of the drug, whether crystalline, amorphous, or metastable, plays a critical role in the stability and performance of the resulting nanoparticles.^36,57^

## 4 Conclusions

This systematic comparison of SNaP and FNP reveals fundamental differences between these nanoprecipitation techniques that significantly impact nanoparticle formation, drug encapsulation, and release behavior. Our investigation demonstrates that while both methods follow concentration-dependent growth mechanisms, they differ substantially in their assembly dynamics and subsequent particle characteristics.

SNaP offers a broader size tunability range compared to FNP across identical total solids concentrations and enables stable nanoparticle formation at dilute conditions where FNP showed limitations. This enhanced versatility stems from SNaP’s decoupled assembly mechanism, allowing core components to assemble before stabilizer addition.

Our drug encapsulation studies across a hydrophobicity spectrum reveal complementary performance profiles between the two techniques. Both methods efficiently encapsulate highly hydrophobic drugs like β-carotene (Log *P* 13.5), while SNaP demonstrates advantages for moderately hydrophobic compounds. For cinnarizine (Log *P* 5.7), SNaP achieved 35% encapsulation efficiency with stable nanoparticles, whereas FNP produced unstable aggregates under the tested conditions. Similarly, with ibuprofen (Log *P* 3.5), SNaP formed stable nanoparticles with 14.4% encapsulation efficiency while FNP did not. These results establish SNaP as the preferred method for encapsulating compounds with moderate hydrophobicity.

The itraconazole release studies reveal another critical advantage of SNaP—enhanced control over drug release kinetics. SNaP-produced nanoparticles exhibited approximately 50% slower release rates compared to FNP formulations over 48 h, suggesting distinct differences in core organization despite identical material composition. This slower release profile, combined with greater encapsulation efficiency, positions SNaP as particularly valuable for controlled-release applications.

Together, these findings establish SNaP as a versatile platform technology that overcomes key limitations of traditional FNP. By decoupling core formation from stabilization, SNaP expands the range of formulation options available to pharmaceutical scientists—offering additional size control capabilities, improved encapsulation of moderately hydrophobic drugs, and enhanced control over release kinetics. As nanomedicine research advances toward clinical applications, SNaP’s ability to address challenges in polymeric nanoparticle formulation makes it a promising addition to the nanoprecipitation toolkit for developing next-generation drug delivery systems.

## Supplementary Material



## Notes

Abbreviations list, HPLC calibration curve data, a comparison of FNP methodology, drug supersaturation calculations, additional itraconazole SNaP nanoparticle data (PDF)
