# Supplementary material for: A Comparative Study of Flash Nanoprecipitation and Sequential Nanoprecipitation: Impact of Formulation Parameters on Drug-Loaded Nanoparticle Formation
Source: Mol Pharm. 2025 Sep 17;22(10):6108–19. doi: 10.1021/acs.molpharmaceut.5c00835 (PMC12505255; doi:10.1021/acs.molpharmaceut.5c00835)
Supplement: Supplementary file 1 [file mp5c00835_si_001.pdf]

Supporting Information

A comparative study of flash nanoprecipitation and sequential nanoprecipitation: Impact of formulation parameters on drug-loaded nanoparticle formation

Nouha El Amri[1], Amy McKinstry[1], Rachel E. Pollard[2], Parker K. Lewis[1], Nathalie M. Pinkerton[1,2‡]

[1]Department of Chemical and Biomolecular Engineering, Tandon School of Engineering, New York University, NY, 11201, USA

[2]Department of Biomedical Engineering, Tandon School of Engineering, New York University, NY, 11201, USA

‡ Corresponding author email: Nathalie.Pinkerton@NYU.edu

***List of Abbreviations*:**

- BCP: Block Copolymer
- CIJM: Confined Impinging Jet Mixers
- DL: Drug Loading
- DLS: Dynamic Light Scattering
- EE: Encapsulation Efficiency
- FNP: Flash NanoPrecipitation
- HIP: Hydrophobic Ion Pairing
- HPLC: High Performance Liquid Chromatography
- MIVM: Multi-Inlet Vortex Mixer
- NP: Nanoparticle
- PDI: Polydispersity Index
- PLA-PEG: Poly (Lactic acid)–Poly (Ethylene Glycol)
- PLA: Poly (Lactic acid)
- SEM: Scanning Electron Microscopy
- SNaP: Sequential NanoPrecipitation
- TEM: Transmission Electron Microscopy
- TGA: ThermoGravimetric Analyzer

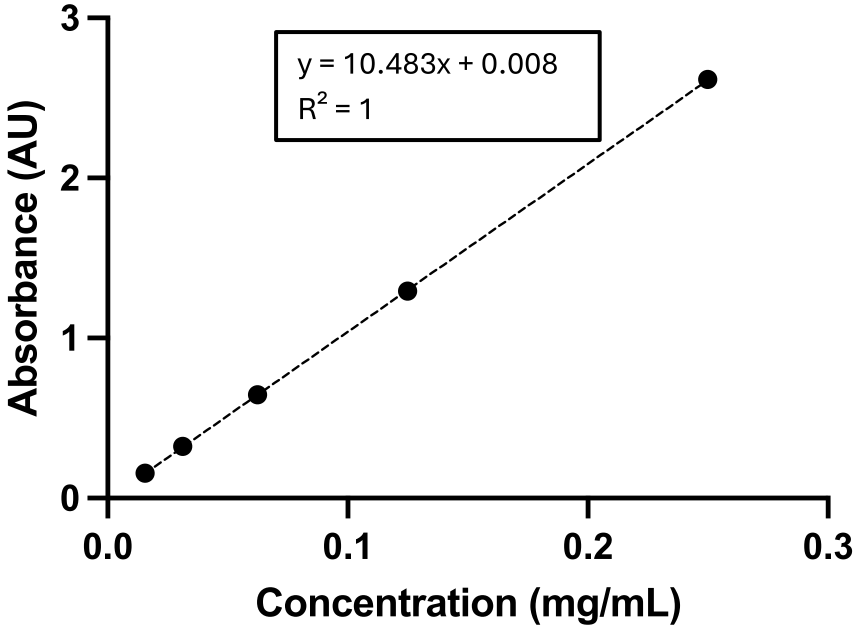

Figure S1: Absorbance calibration curve of β-carotene

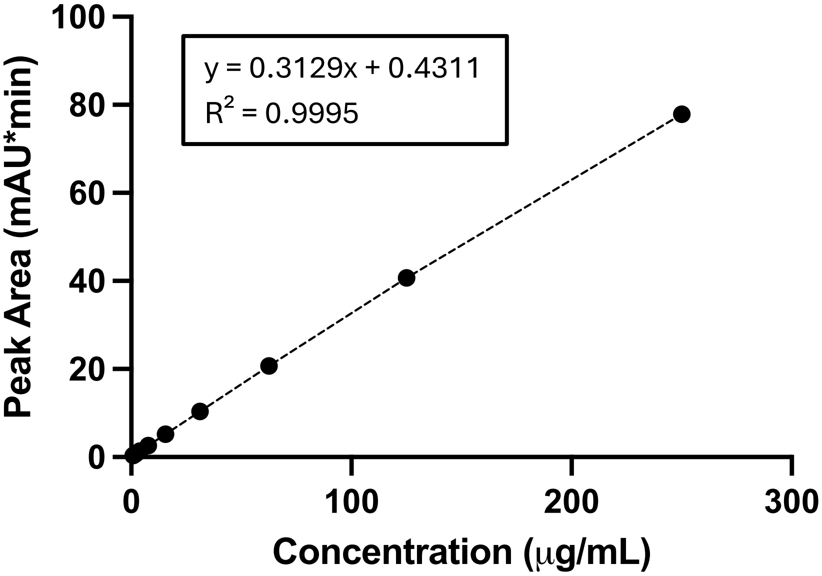

Figure S2: High Performance Liquid Chromatography (HPLC) calibration curve of ibuprofen.

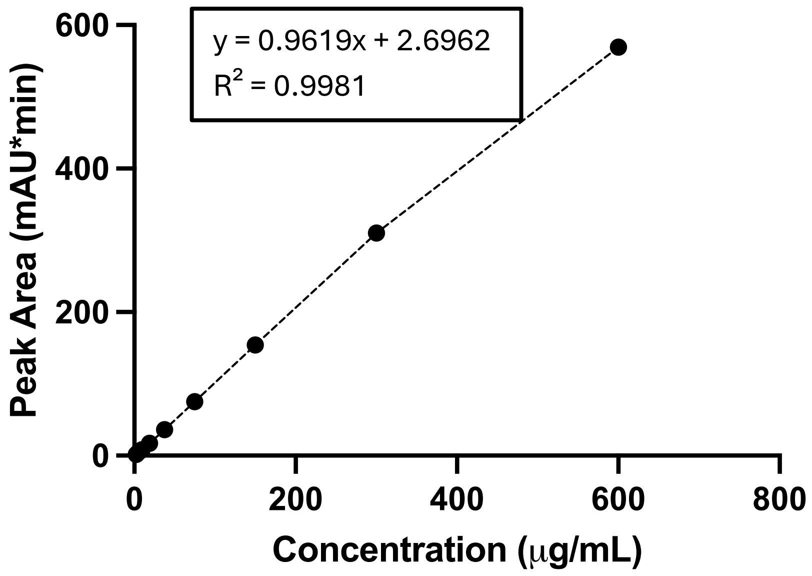

Figure S3: High Performance Liquid Chromatography (HPLC) calibration curve of cinnarizine.

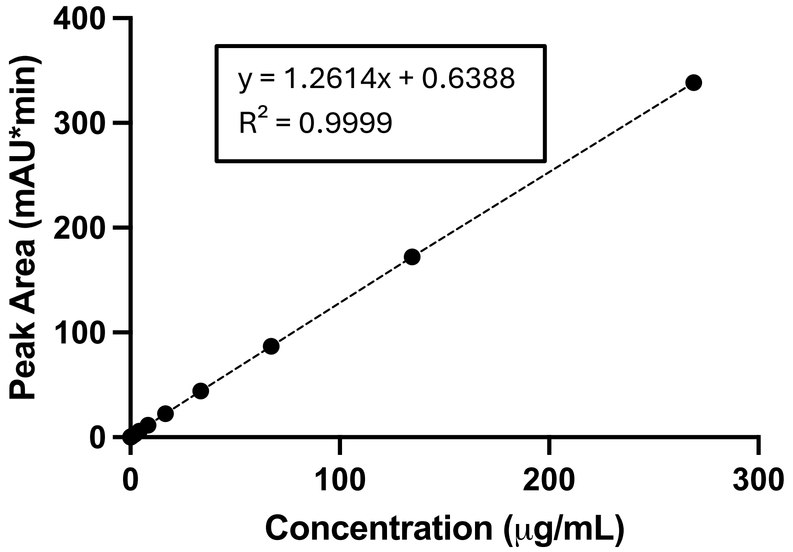

Figure S4: High Performance Liquid Chromatography (HPLC) calibration curve of itraconazole.

Comparison of FNP methodology

To validate the robustness of the manual FNP plunge method, we performed additional FNP beta-carotene nanoparticle syntheses in triplicate using syringe pumps instead of the manual plunge. The resulting nanoparticles had a similar size as shown in the DLS trace below. The manually depressed nanoparticles from the main text had an average size of 53 ± 9 nm, while the syringe pump nanoparticles shown here had an average size of 61 ± 8 nm. The drug loading and encapsulation efficiency of the manually depressed nanoparticles was 19 ± 2% and 97 ± 8%, while that of the syringe pump nanoparticles was 20 ± 1 % and 104 ± 8%. No significant difference was observed between the two methods.

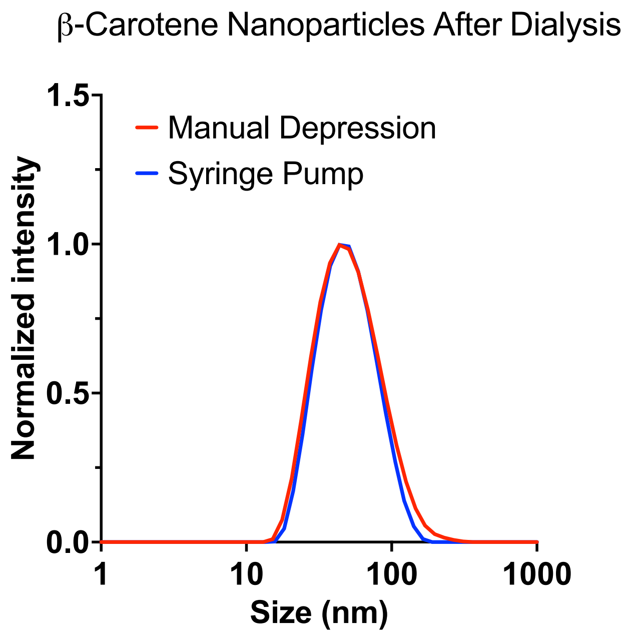

Figure S5: Intensity weighted DLS traces of β-carotene-loaded PEG-PLA nanoparticles formed via FNP using either the manual depression method or a syringe pump at a flow rate of 35 mL/min per stream after dialysis.

Calculation of Drug Supersaturations

To estimate the supersaturation of each of the drugs, equation 4 from the main text was used:

$$S = \frac{C}{C_{eq}}$$

Where S is the supersaturation, C is the drug solution concentration and C_{eq} is the equilibrium drug concentration.[1] Because the C_{eq} of the drugs in the solvent conditions at the point of mixing (25/75 vol/vol THF/water) were not readily available in the literature, we instead used the C_{eq} values of the drugs in water. The values and resulting supersaturations are shown in Table S1 below.

Table S1. Estimated drug supersaturation values

**Drug**	**LogP**	**C (mg/ml)**	**C_{eq} (mg/mL)**	**S**
β-carotene	13.5	1.50	0.000391[a]	3840
Cinnarizine	5.7	0.38	0.002[b]	190
Itraconazole	5.6	0.75	0.00964[a]	80
Ibuprofen	3.5	1.13	0.0684[a]	20

a. Data from go.drugbank.com accessed on 21 August 2025
b. Data from Shakeel F, Kazi M, Alanazi FK, Alam P. Solubility of Cinnarizine in (Transcutol + Water) Mixtures: Determination, Hansen Solubility Parameters, Correlation, and Thermodynamics. Molecules. 2021 Nov 22;26(22):7052. doi: 10.3390/molecules26227052.

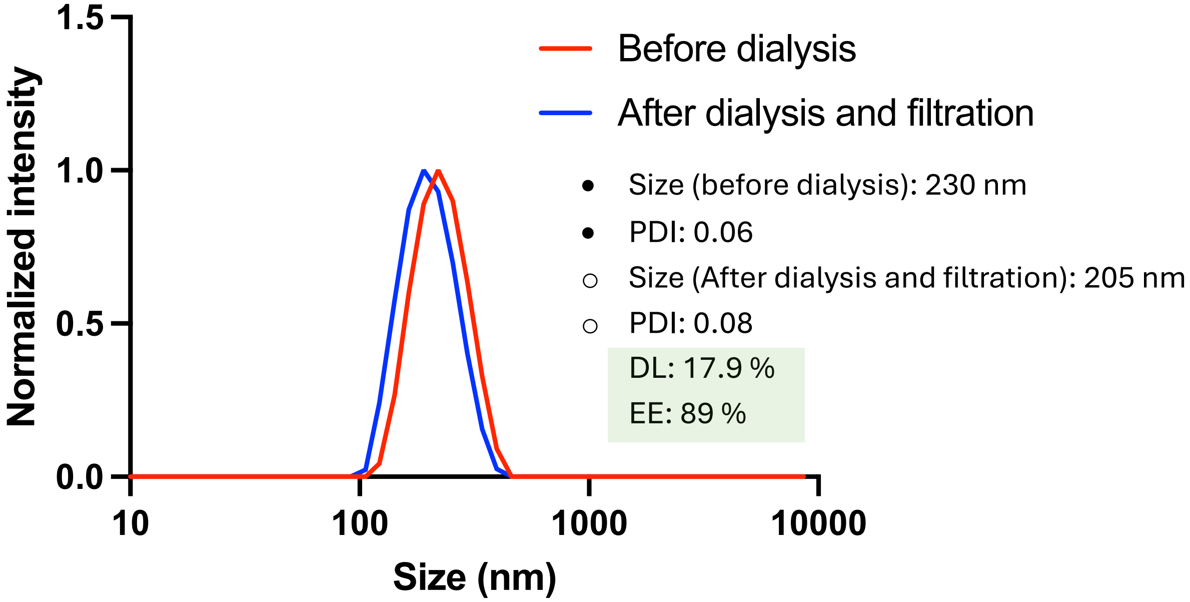

Figure S5: Intensity weighted DLS trace of Itraconazole-loaded PEG-PLA nanoparticles formed via SNaP (20% loading) before and after dialysis and filtration.

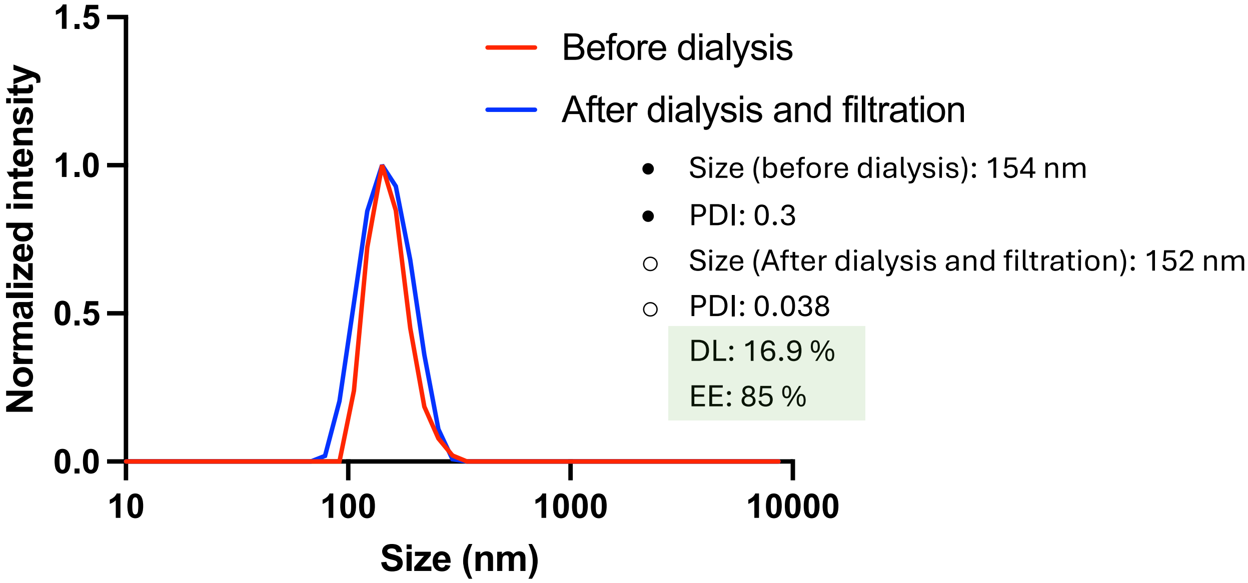

Figure S6: Intensity weighted DLS trace of Itraconazole-loaded PEG-PLA nanoparticles formed via FNP (20% loading) before and after dialysis and filtration.

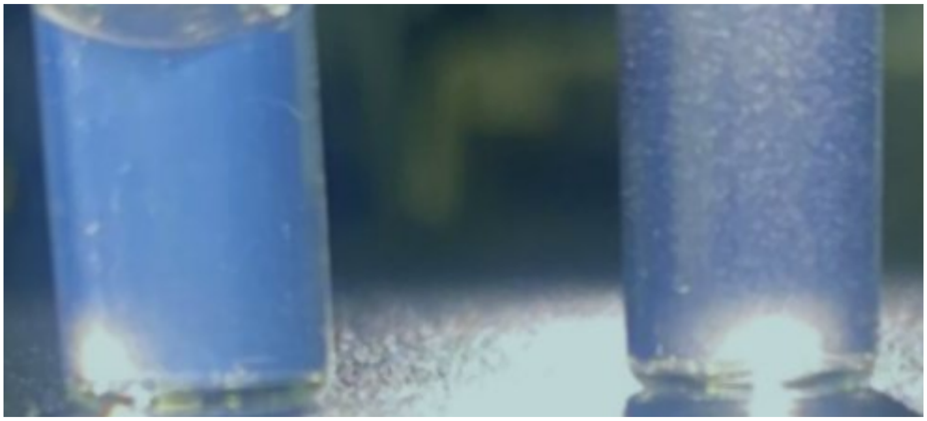

Figure S7: A photo of the Itraconazole-loaded PEG-PLA nanoparticles (20% loading) dispersion formed via FNP (right) and SNaP (left) after 24h. This photo shows that the FNP sample was not stable, as evidenced by the visible precipitate. In contrast, the SNaP sample was stable (no visible precipitate was observed even after 2 weeks of the synthesis).

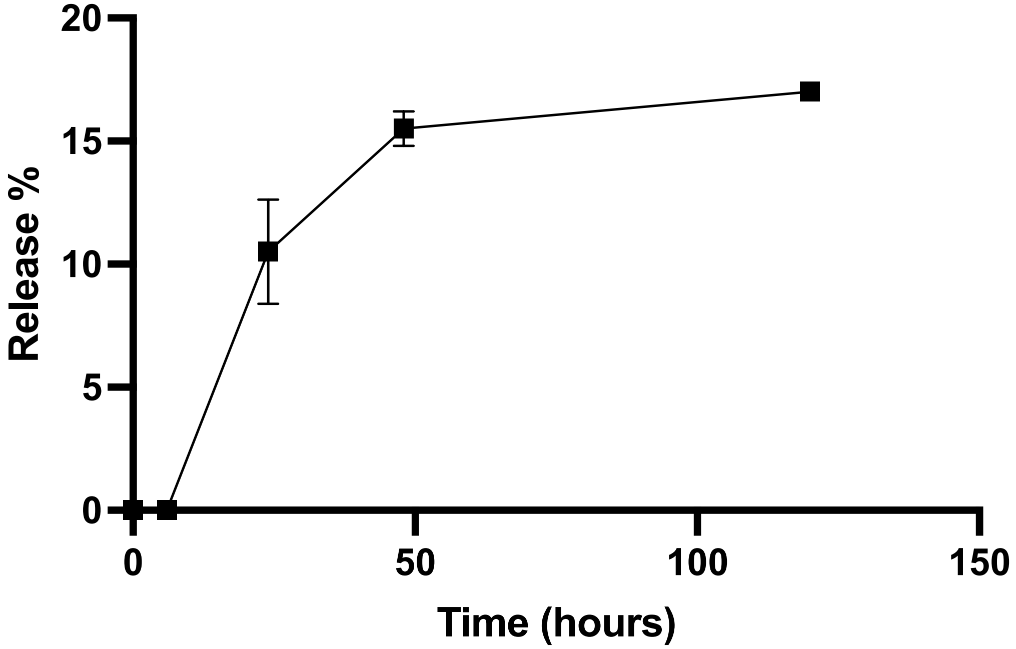

Figure S8: Drug-release profile of itraconazole-loaded nanoparticles (with 20% loading) formed via SNaP over 120 hours (in PBS, pH 7.4, 37°C, and 5% Tween).

References

(1) Brick, M. C.; Palmer, H. J.; Whitesides, T. H. Formation of colloidal dispersions of organic materials in aqueous media by solvent shifting. Langmuir **2003**, 19 (16), 6367-6380.
